# Association between Markers of Fibrosis and Heart Failure Incidence in Patients with Type 2 Diabetes Mellitus

**DOI:** 10.1155/2021/9589185

**Published:** 2021-11-05

**Authors:** Denis A. Lebedev, Elena A. Lyasnikova, Elena Yu. Vasilyeva, Nikolai P. Likhonosov, Maria Yu. Sitnikova, Alina Yu. Babenko

**Affiliations:** ^1^Almazov National Medical Research Centre, Saint Petersburg, Russia; ^2^St. Petersburg State University Hospital, Saint Petersburg, Russia

## Abstract

Type 2 diabetes mellitus (T2DM) and chronic heart failure (HF) have close association, and several biomarkers have been studied to better understand this association and improve prediction of HF in T2DM. Furthermore, in recent clinical trials, sodium glucose cotransporter 2 inhibitors (SGLT2i), glucose-lowering drugs, improved HF outcomes. The objective of the present study was to evaluate association between circulating biomarkers of fibrosis and incidence of HF with preserved ejection fraction (HFpEF) in patients with T2DM receiving sodium glucose cotransporter 2 inhibitors (SGLT2i). *Materials and Methods*. At baseline, transthoracic echocardiography and laboratory assessment of N-terminal fragment of the brain natriuretic peptide (Nt-proBNP), soluble suppression of tumorigenesis-2 (sST2), galectin-3 (Gal-3), C-terminal propeptide of procollagen type I (PICP), N-terminal propeptide of procollagen type III (PIIINP), matrix metalloproteinase-9 (MMP-9), and tissue inhibitor of matrix proteinase-1 (TIMP-1) were done. After 3 years of follow-up, information about HF events (hospitalization for HF, established HF in outpatient department by a cardiologist) was obtained. *Results*. Seventy-two patients were included in the study. The mean age was 57 (49.7; 63.2) years; 44% were female. Most patients had T2DM for more than 4 years. All patients were overweight or had obesity, and 93% patients had arterial hypertension (AH). After 3 years of follow-up, HFpEF was established in 21% patients. Patients were divided into two groups according to the presence of HFpEF, and baseline characteristics were compared. Patients with HF were older and had longer diabetes and AH duration and higher Nt-proBNP, Gal-3, PIIINP, and PICP levels at baseline than patients without HF (all *p* < 0.05). Gal − 3 > 10 ng/ml (OR = 2.25; 95% CI, 1.88–5.66; *p* = 0.01) and NT − pro − BNP > 80 pg/ml (OR = 2.64; 95% CI, 1.56–4.44; *p* = 0.001) were associated with increased risk of HF incidence. Age > 60 years, diabetes duration > 10 years, and presence of abdominal obesity were independent predictors of HFpEF as well. *Conclusions*. T2DM patients treated with SLGT2i, who developed HFpEF after 3 years of follow-up, had higher PICP, PIIINP, Gal-3, and NT-proBNP serum concentrations at baseline, and Gal-3 level was an independent predictor of HFpEF.

## 1. Introduction

Type 2 diabetes mellitus (T2DM) is one of the most important chronic conditions nowadays, which is tightly linked to development of chronic heart failure (HF). The prevalence of HF is 4 times higher in patients with T2DM than in the general population [1]. T2DM can contribute to HF via different mechanisms such as low-grade inflammation, oxidative stress, endothelial dysfunction, and fibrosis [2]. These processes lead to diabetic cardiomyopathy, acceleration of atherosclerosis, increased arterial stiffness, and myocardial ischemia. Several biomarkers have been studied in order to better understand HF development in T2DM and improve the prediction of HF incidence and its progression [3, 4]. N-terminal pro-B-type natriuretic peptide (NT-proBNP) was identified as a reliable marker for HF [1]. The predictive value of circulating biomarkers of fibrosis in a HF incidence and T2DM-induced cardiomyopathy was shown in several studies and still being discussed and studied in patients with T2DM [5]. In some studies, there was an association of type 1 collagen degradation products and different phenotypes of HF in patients with T2DM [4, 6]. Specific scales for predicting HF in T2DM patients are being actively developed, and it is assumed that those markers that are specifically elevated in T2DM patients with HF may be elevated also in patients with T2DM who will develop HF in the near future. Although biomarkers can improve management of HF, there is no clear data regarding cost-effectiveness for each biomarker in clinical practice.

Moreover, there is therapy, which is useful in patients with T2DM and HF. A number of studies have shown that sodium glucose cotransporter 2 inhibitors (SGLT2i) significantly reduce the risk of HF in patients with T2DM [7, 8]. SGLT2i are recommended in patients with T2DM at high risk of CV events or with CV disease to reduce hospitalizations for HF, major CV events, and CV death [9]. Along with metabolic and hemodynamic protective mechanisms, SGLT2i exhibit anti-inflammatory, antiapoptotic, and antifibrotic effects [10]. Furthermore, both animal and clinical studies have demonstrated an inhibitory effect on sympathetic nerve activation [10]. Although beneficial effects of SGLT2i were demonstrated, there is not much data regarding prognostically relevant biomarkers of fibrosis among patients with T2DM treated with SGLT2i. The aim of this study was to assess the relationship between circulating biomarkers of fibrosis and the incidence of HFpEF in T2DM patients receiving SGLT2i.

## 2. Materials and Methods

**Figure 1:**
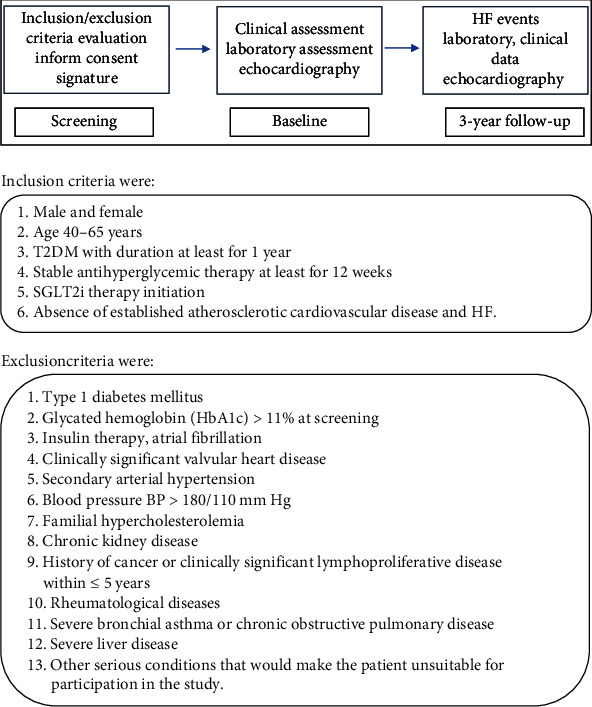
Study design.

We conducted a prospective study in the T2DM patient's population. The study was conducted at Almazov National Medical Research Centre. The Ethical Committee of the Almazov National Medical Research Centre approved the study, and procedures were done in compliance with the ethical principles outlined in the Declaration of Helsinki and ICH E6 (R2) good clinical practice. The study outcome was incidence of HF. Clinical, laboratory, and instrumental data were collected at baseline. After 3 years of follow-up, information about HF events (hospitalization for HF, established HF in outpatient department by a cardiologist) and other clinically important data were obtained from medical records. Also, at the end of the study echocardiography, HbA1c, fasting lipids, and creatinine were evaluated. The study design schematic is shown in Figure 1.

Demographic information such as age, sex, weight, height, body mass index (BMI), waist circumference (WC), medical history, and duration of diabetes were collected from patients' medical records. Abdominal obesity was defined as a waist circumference of more than 88 cm in women and more than 102 cm in men. Twelve-lead electrocardiogram (ECG) and transthoracic echocardiogram were obtained at baseline. Cardiovascular disease and HF were excluded by the cardiologist. Echocardiography (VIVID 9 GE, USA) was performed according to the standard protocol by one operator. Left atrial volume (LAV) and left ventricular myocardial mass (LVMM) were indexed to body surface area (BSA) and indexed to height with various allometric powers.

Blood samples were drawn from each subject after fasting for at least 8 h. HbA1c, fasting lipids, creatinine, and transaminases were measured. The estimated glomerular filtration rate (eGFR) was calculated using the Chronic Kidney Disease Epidemiology Collaboration equation (CKD-EPI). The serum samples for biomarkers were frozen at -80°C until analysis. С-reactive protein (CRP), NT-proBNP, sST2 (stimulating factor growth expression gene 2, soluble form, also known as IL1RL1, and suppression of tumorigenicity 2), galectin-3 (Gal-3), type I procollagen C-terminal propeptide (PICP) and type III procollagen N-terminal propeptide (PIIINP), matrix metalloproteinase (MMP-9), and tissue inhibitor of matrix-metalloproteinase (TIMP-1) were measured in serum. Nt-proBNP level was assessed by an electrochemiluminescence method. For assessment of PICP, PIIINP, Gal-3, MMP-9, and TIMP-1, enzyme immunoassay was used. For Nt-proBNP measurement, Elecsys test system (Roche Diagnostic) was used. For Gal-3 MMP-9 and TIMP1, R&D system kits were used; sST2 was measured by clinical diagnostics, Presage ST2 kit, and PICP and PIIINP by USCN Life Science kits.

### 2.1. Statistical Analysis

The data were evaluated using the IBM SPSS statistical software (version 21.0, IBM Corp, USA). Continuous variables are expressed as median (interquartile range), and categorical variables are expressed as number (percentage). Differences between groups were tested using the Mann–Whitney *U* test. Categorical variables were compared by chi-squared test. ROC analysis was done for biomarkers. In order to describe relative risk, the odds ratio (OR), with 95% confidence interval (95% CI), was calculated. Logistic regression was performed to identify risk factors for HF. All demographic and clinical characteristics were investigated as potential predictors. Firstly, candidate variables were analyzed in univariate models. If the *p* value was less than 0.1, the respective variable was included in a multivariable logistic regression model. Statistical significance was defined as a *p* value < 0.05.

## 3. Results and Discussion

### 3.1. Baseline Information

Seventy-two patients were included in the present study.  Table 1 shows the baseline characteristics of study participants. All patients had T2DM and were not experienced any cardiovascular events. The mean age was 57 (49.7; 63.2) years; 44.4% were female. Most patients had T2DM for more than 4 years. All patients were overweight or had obesity, and 67 (93%) patients had arterial hypertension controlled by medications. Forty-nine (68%) patients received statins, and mean LDL was 2.68 mmol/l (1.70; 3.39). Mean HbA1c was 8.4% (7.8; 9.2); all patients received oral antihyperglycemic medications. Empagliflozin 10 mg per day was prescribed for all patients.

### 3.2. Clinical Outcome and Comparison of HF and Non-HF Groups

After 3 years of follow-up, HFpEF was established in 15 patients. Two myocardial infarctions occurred in the non-HF group and one in HF group. Patients were divided into two groups according to the presence of HFpEF. Baseline data were compared between these two groups. Results of this comparison are presented in Table 1. Patients with HFpEF were older than patients without HFpEF and had longer diabetes and arterial hypertension duration. There were no differences in gender, history of smoking, systolic and diastolic office blood pressure, BMI, HbA1c, and glucose levels. The HF group had higher waist circumference values and abdominal obesity compared with the non-HF group (all *p* < 0.05). HF patients had higher Nt-proBNP levels at baseline than patients without HF (*p* = 0.001). HF patients had higher Gal-3 levels at baseline than patients without HF (*p* = 0.012). The same situation was observed for PIIINP, concentrations of this biomarker were higher in the HF group (*p* = 0.033). Patients with HFpEF had higher levels of PICP compared with non-HF patients, 137 ng/ml (116.3; 175.5) and 115.2 ng/ml (71.8; 152.6), respectively, *p* = 0.026. There were no significant differences in baseline therapy for AH and T2DM, statins, levels of GFR, and concentrations of LDL, HDL, and TG between two groups as well as in the baseline myocardial morphofunctional parameters (all *p* > 0.05). At the same time, there was also difference in duration of SGLT2i therapy. Patients in the HF group less likely received empagliflozin for more than 1 year than patients in the non-HF group—40% and 71.9%, respectively, *p* = 0.01.

### 3.3. Risk Factors for HF Incidence

**Figure 2:**
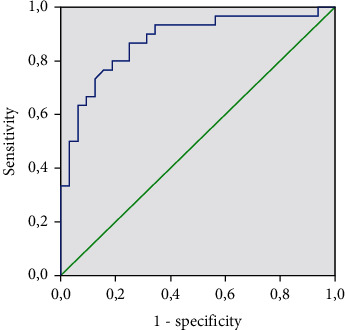
Galectin-3 level and HFpEF incidence.

To assess the predictive value of Gal-3 and find the optimal classification threshold, a ROC analysis was performed. The threshold for Gal-3 level associated with increased risk of HFpEF in this population was 10.25 ng/ml (AUC area = 0.876; sensitivity, 86%; and specificity, 72%; *p* < 0.001) (Figure 2).

**Figure 3:**
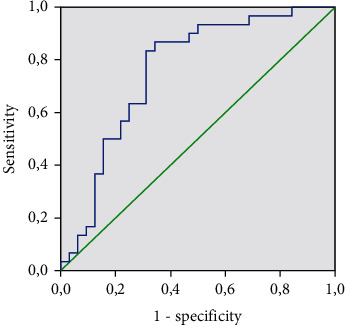
NT-proBNP level and HFpEF incidence.

The threshold for NT-proBNP was 77.55 pg/ml (AUC area = 0.757; sensitivity, 83%; and specificity, 69%; *p* = 0.001) (Figure 3).

Multiple logistic regression analysis identified significant risk factors for new onset of HFpEF (Table 2). Age > 60 years, diabetes duration > 10 years, and presence of abdominal obesity were independent predictors of HFpEF. The most significant factor was NT-pro-BNP level > 80 pg/ml (OR = 2.64; 95% CI, 1.56–4.44; *p* = 0.001). Gal-3 level > 10 ng/ml was associated with increased risk of HF incidence (OR = 2.25; 95% CI, 1.88–5.66; *p* = 0.01). Whereas every unit rise in Gal-3 more than 10 ng/ml increased the risk for new-onset HFpEF by about 25%, a unit increase in NT-pro-BNP more than 80 pg/ml increased the odds by about 64%. Other markers of fibrosis were not significant risk factors for incident HF as well as sex, duration of AH, and echocardiographic parameters.

Table 3 shows comparison of results, obtained after 3 years of follow-up. When echocardiography results after 3 years of follow-up between studied groups were compared, patients in the HF group had higher left atrial volume index (LA volume index) than the non-HF group. Relative wall thickness of the left ventricle (RWT), LVM/BSA, and LVM/height^2,7^ were also significantly higher in patients with HFpEF. There were no differences in GFR, LDL, HDL, and TG levels between groups. However, HbA1c levels were significantly lower in the non-HF group.

## 4. Discussion

Twenty-one percent of patients in our study developed HFpEF after 3 years of follow-up. These patients had clinical (age, abdominal obesity, duration of T2DM, and arterial hypertension) and laboratory risk factors associated with HF incident. Obesity is a well-known risk factor for HF, and visceral adiposity can be possibly related for this link [11]. In our study, WC as a marker of abdominal obesity was significantly higher in patients developed HF. Indeed, it was reported that in patients with T2DM, excessive visceral fat has a stronger association with the development of LV diastolic dysfunction than glycemic control [12]. An increase in T2DM duration is also associated with an increased risk for HF [13]. Thus, our results are consistent with the data from other studies, and the ongoing therapy with SGLT2 inhibitors did not change this association. Although not all patients took SLGT2i for 3 years, the percentage of patients in the HF group who were treated for more than a year was lower than that in the non-HF group, which could affect the outcome.

It is well established that cardiac fibrosis is associated with HF. As the result of the predominance of the synthesis of type I and III collagen over their degradation, the excess of collagen type I and III fibers is being accumulated within the myocardium [14]. There is a wide spectrum of biomarkers, which reflects several stages of TDM pathogenesis and could predict CV risk [6, 15]. In the present study, the patients with a developed HFpEF after 3 years of follow-up had elevated PICP, the marker of collagen type I synthesis, and PIIINP, the marker of collagen type III synthesis. Previous studies have shown that serum PICP concentrations are increased in hypertensive patients and have strong correlation with myocardial collagen content [16]. Furthermore, in patients with HF and preserved EF, plasma levels of procollagen type I amino-terminal peptide and procollagen type III amino-terminal peptide were associated with increased mortality and cardiovascular hospitalization [17]. Also, PIIINP and collagen type I carboxy-terminal telopeptide (ICTP), other collagen biomarker, also appeared to be related to incident HFpEF, but not HFrEF [18]. Delicate balance between the synthesis and degradation of two types of collagen can determine the structural and functional changes in the myocardium in HF patients with impaired glycemic status [4]. Our data suggest that PIIINP may be considered a predictor for HFpEF in T2DM patients. However, it is not fully understood whether these circulating markers of collagen synthesis and degradation can be used to prognosticate CV risk in patients with metabolic disease [6]. Therefore, establishing potentially usefulness of PICP and PIIINP to improve prognosis in cardiac diseases associated with HF of requires further investigation, taking into account possible confounders affecting collagen metabolism.

Gal-3, which is secreted by macrophages, has been known for its role in mediating cardiac fibrosis, and some studies already demonstrated that this biomarker could have a prognostic value in HF [19]. However, the predictive value of Gal-3 in relation to other traditional biomarkers in T2DM patients with HF remains ambiguous [20]. Elevated Gal-3 levels were predictors of T2DM-induced cardiomyopathy and associated with diminished global longitudinal strain in diabetics [21]. In our study, Gal-3 was associated with incident HFpEF. This observation is consistent with previous studies. Thus, Gal-3 was associated with risk for incident HF in participants from the Framingham Offspring Cohort [22]. Also, persistently elevated Gal-3 predicts new-onset HF according to results from another study [23]. Gal-3 is associated with diabetes prevalence and incidence, possibly through the inflammatory pathway contributing to *β*-cell fibrosis and impaired insulin secretion [20]. Significant increase of Gal-3 in T2DM patients with and high risk of HF development may reflect essential violations of neurohumoral activity in T2DM. In a recent study, it was demonstrated that Gal-3 is involved in mechanisms of neurohumoral impairment [24]. Gal-3 was also the only biomarker associated with the development of acute ischemic events and heart failure or death in T2DM patients in one study [25]. Furthermore, serum Gal-3 is associated with adverse cardiovascular outcomes in persons with T2DM independent of traditional risk factors [26]. There are data elucidating the possible interrelation between dynamic changes in levels of Gal-3 and CV risk in T2DM patients treated with antidiabetic drugs including SGLT2i [27]. Gal-3 is a biomarker of fibrosis and, thus, may be involved in interstitial atrial remodeling and related to atrial fibrillation [28, 29]. Experimental and clinical studies have demonstrated a sympathetic inhibitory effect that, beyond being associated with the reduction of fibrosis, by itself an important arrhythmogenic substrate, suggested the potential role of SGLT2i in the prevention of any arrhythmic event [30]. It is important to note that in patients of our group with multiple cardiometabolic risk factors and, therefore, high risks of atrial fibrillation, rhythm disturbances were not diagnosed for 3 years of follow-up. Taking into account the above and the data obtained in our work, Gal-3 is a promising biomarker that stratifies patients at risk of CV events including HF in T2DM patients, as emphasized by other authors [6], and requires further study.

Despite the fact that MMP-9 and TIMP-1 are involved in cardiac remodeling, we did not observe significant differences in concentrations of these biomarkers between HF and non-HF groups. It was shown that TIMP-1 levels were related to left ventricular mass and wall thickness and inversely to systolic function [31]. Furthermore, expression of MMP-9 and TIMP-1 genes has been associated with HF [32]. In addition, some authors hypothesized that MMP-9 and TIMP-1 could be used for prognosis of HF outcomes rather than diagnosis in HF [33].

NT-proBNP is widely used for diagnosis and prognosis for all relevant clinical outcomes in HF [34]. In the present study, patients from the HF group also had higher NT-proBNP and NT-proBNP was an independent predictor of incident HF. According to results from EMREROR-reduced trial, higher baseline NT-proBNP concentrations were associated with greater risk for adverse heart failure outcomes, but empagliflozin reduced risk regardless of baseline NT-proBNP concentration [35]. Initial high level of NT-proBNP was associated with an increased risk of cardiovascular death and hospitalizations for HF in T2DM patients, while there was a decrease in risks during therapy with SGLT2i, regardless of NT-proBNP level [36].

There were no differences in ST2 levels between studied groups. This biomarker, as a biomarker of certain inflammatory condition and fibrosis, was recommended by ACC/AHA as a predictor of hospitalization and death in patients with HF [37]. However, previous studies have reported that sST2 has a weaker predictive value than NT-proBNP in the diagnosis of HF [38]. Furthermore, sST2 levels were not significantly changed in T2DM patients without known HF during long-term treatment with SGLT2i despite improvement in clinical outcomes [39]. Thus, the role and predictive value of sST2 in T2DM are controversial and require further investigation [6].

There are several limitations to our study. Firstly, there was a relatively small sample size and there was significant difference in age between groups. Secondly, markers of fibrosis are not absolutely specific for cardiac fibrosis and presence of confounding factors can have influence on the studied biomarkers. In addition, there were no serial measurements of these biomarkers in our study and not all patients received SGLT2i for 3 years.

## 5. Conclusions

T2DM patients treated with SLGT2i, who developed HFpEF after 3 years of follow-up, had higher PICP, PIIINP, Gal-3, and NT-proBNP serum concentrations at baseline, and Gal-3 level was an independent predictor of HFpEF. Predictive value needs to be clarified for some biomarkers of fibrosis in T2DM in future studies taking into account the economic aspects it is using. The research on large samples is required to identify T2DM patients at high risk for the development of HFpEF, based on individual risk profiles for targeted prevention and treatment.

## Figures and Tables

**Table 1:** Baseline clinical characteristics and echocardiographic and biomarker data of study population, Me (25; 75), *n* (%).

Parameters	All patients	Non-HF (*n* = 57)	HF (*n* = 15)	*p* (for HF and non-HF group)
Age (years)	57 (49.7; 63.2)	52.5 (45; 61)	60.5 (54.5; 66)	0.005
Gender, female, *n* (%)	32 (44.4)	25 (43.8)	7 (46.6)	0.32
Diabetes duration, years, *n* (%)	8 (4.7; 12.2)	5.5 (3.2; 8.0)	11.5 (8.2; 16)	<0.001
Arterial hypertension, *n* (%)	67 (93%)	53 (92.9)	14 (93.3)	<0.001
Arterial hypertension duration (years), *n* (%)	10.2 (4.3; 15.9)	7.5 (3.8; 12.4)	10.1 (6.5; 16.2)	<0.001
Smoking, *n* (%)	31 (43)	25 (43.8)	6 (40)	0.26
BMI (kg/m^2^)	33.4 (30.5; 35.8)	33.1 (30.2; 34.8)	34.5 (30.9; 38.4)	0.08
WC (cm)	107.5 (98.7; 114.2)	103 (95.2; 109.7)	109 (101.7; 121.5)	0.019
Abdominal obesity, *n* (%)	45 (62.5)	33 (57.8)	12 (80)	0.035
Systolic BP (mmHg)	134 (99; 146)	130 (94; 142)	136 (97; 147)	0.43
Diastolic BP (mmHg)	94 (72; 106)	93 (75; 101)	90 (80; 109)	0.27
Glucose fasting (mmol/l)	9.2 (8.2; 10.5)	9.1 (7.5; 10.4)	9.3 (8.6; 11.1)	0.26
HbA1c (%)	8.4 (7.8; 9.2)	8.2 (7.5; 9.0)	8.5 (7.9; 9.5)	0.39
Metformin, *n* (%)	61 (84.7)	49 (85.9)	13 (8.6)	0.75
DPPi-4, *n* (%)	29 (40.3)	30 (52.6)	7 (46.6)	0.34
Sulfonylurea, *n* (%)	20 (27.7)	14 (17.5)	4 (26.6)	0.41
ARA/ACEi, *n* (%)	56 (77.7)	44 (77.2)	10 (66.6)	0.28
Calcium channel blockers, *n* (%)	31 (43.1)	24 (42.1)	8 (53.3)	0.12
Diuretics, *n* (%) Statins, *n* (%)	24 (33.3) 49 (68)	15 (26.3) 39 (68.4)	5 (33.3) 10 (66.6)	0.21 0.71
CRP (mg/l)	2.69 (1.15; 5.6)	2.06 (0.87; 5.27)	3.35 (2.04; 5.7)	0.19
NT-pro-BNP (pg/ml)	72.78 (43.34; 96.2)	46.45 (19.81; 88.35)	103.4 (80.1; 118.3)	0.001
ST2 (ng/ml)	22.2 (17.5; 26.4)	22.97 (16.96; 27.98)	23.78 (17.45; 29.21)	0.62
Galectin-3 (ng/ml)	10.7 (8.0; 13.3)	9.82 (7.46; 12.19)	12.64 (9.22; 14.95)	0.012
PICP (ng/ml)	130.4 (101.3; 159.8)	115.2 (71.8; 152.6)	137 (116.3; 175.5)	0.026
PIIINP (ng/ml)	7.05 (3.6; 17.4)	4.36 (3.36; 12.99)	10.56 (9.22; 14.95)	0.033
PICP/PIIINP	19.3 (10.5; 34.2)	32.6 (15.4; 41.6)	10.8 (4.9; 22.7)	0.001
MMP-9 (ng/ml)	527.4 (345.2; 749.7)	433.1 (184; 648.7)	568.5 (200.6; 823.45)	0.051
TIMP-1 (ng/ml)	204 (168.5; 272.6)	213.5 (174.7; 278.3)	193.5 (128.5; 255.1)	0.12
MMP-9/TIMP-1	2.2 (1.6; 3.9)	2.4 (1.4; 4.2)	1.9 (0.9; 3.2)	0.18
GFR (ml/min/1.73 m^2^)	78.5 (71; 87.2)	73.5 (68; 84)	80 (74; 91)	0.15
LDL (mmol/l)	2.68 (1.70; 3.39)	2.87 (2.06; 3.81)	2.3 (1.43; 3.14)	0.31
HDL (mmol/l)	1.03 (0.89; 1.22)	1.08 (0.89; 1.23)	1.0 (0.88; 1.23)	0.78
TG (mmol/l)	2.28 (1.79; 3.02)	2.5 (1.9; 3.2)	2.08 (1.64; 2.97)	0.14
EF (Simpson) (%)	62 (58; 64)	62 (55; 66)	58 (49; 63)	0.24
LA volume index (ml/m^2^)	34.2 (30.4; 38.7)	38.3 (32.4; 42.1)	41.8 (34.2; 45.8)	0.15
*E*/*e*	9 (7; 10)	8 (7; 9)	10 (8; 12)	0.06
IVS (mm)	10 (9; 12)	10 (9; 13)	11 (10; 13)	0.63
PW (mm)	11 (10; 13)	10 (9; 12)	11 (10; 13)	0.68
RWT	0.46 (0.41; 0.49)	0.46 (0.43, 0.52)	0.49 (0.42; 0.56)	0.31
LV EDV (ml)	110 (95.5; 118.3)	115 (98.2; 128.9)	120.5 (105; 138.4)	0.22
LV ESV (ml)	48 (37; 56)	44 (35; 50)	48 (40; 56)	0.39
LVM/BSA (g/m^2^)	109 (96; 117)	109 (94; 129)	120 (98; 140)	0.47
LVM/height (g/m^2.7^)	50 (46; 59)	45 (40.3; 51.4)	47.9 (42.3; 53.4)	0.29

BMI: body mass index; BP: blood pressure; WC: waist circumference; GFR: glomerular filtration rate; ACEi: angiotensin-converting-enzyme inhibitors; ARA: angiotensin receptor antagonists; DPPi-4: dipeptidyl peptidase-4 inhibitors, LDL: low-density lipoprotein; HDL: high-density lipoprotein; TG: triglycerides; EF: ejection fraction; LA: left atrium; LV: left ventricle; ESV: end-systolic volume of the left ventricle; EDV: end-diastolic volume of the left ventricle; PW: posterior wall of left ventricle; RWT: relative wall thickness of the left ventricle; LVM: left ventricular myocardial mass; BSA: body surface area.

**Table 2:** Multiple logistic regression analysis for HFpEF incidence.

Predictor	OR (95% CI)	*p*
Age > 60 (years)	1.60 (1.11-2.87)	0.015
Diabetes duration > 10 years	1.56 (1.23-2.21)	0.021
Abdominal obesity	1.38 (1.09-2.22)	0.027
Galectin − 3 > 10 ng/ml	2.25 (1.88–5.66)	0.006
NT − pro − BNP > 80 (pg/ml)	2.64 (1.56-4.44)	0.001

Data are shown as odds ratios (OR) together with the 95% confidence interval (CI) and the corresponding *p* value.

**Table 3:** Comparison of echocardiography and laboratory results between groups, Me (25; 75).

Parameters	Non-HF (57)	HF (15)	*p*
HbA1c (%)	7.5 (7.2; 8.1)	8.2 (7.4; 9.6)	0.035
GFR (ml/min/1.73 m^2^)	77 (66; 89)	71 (65; 84)	0.41
LDL (mmol/l)	2.67 (2.16; 3.31)	2.54 (1.48; 3.22)	0.29
HDL (mmol/l)	1.02 (0.85; 1.14)	0.97 (0.89; 1.08)	0.73
TG (mmol/l)	2.7 (1.6; 3.8)	2.9 (1.9; 4.0)	0.22
EF (Simpson) (%)	62 (56; 65)	59 (55; 63)	0.56
LA volume index (ml/m^2^)	36.3 (31.8; 40.1)	43.9 (37.7; 46.3)	0.008
IVS (mm)	10 (9; 12)	11 (10; 13)	0.64
PW (mm)	10 (9; 12)	11 (9; 13)	0.56
RWT	0.46 (0.42; 0.49)	0.55 (0.48; 0.59)	0.016
LV EDV (ml)	118 (101.2; 128.9)	125.5 (115.3; 139.5)	0.38
LV ESV (ml)	45 (38; 51)	46 (42; 53)	0.42
LVM/BSA (g/m^2^)	108 (90; 116)	120 (99; 139)	0.026
LVM/height (g/m^2.7^)	49 (41; 58)	59 (50; 70)	0.031

## Data Availability

Research data can be available after contact by e-mail (doctorlebedev11@gmail.com) to the corresponding author.
